# Onychopathy Induced by Nivolumab: A Targeted Immunotherapy

**DOI:** 10.7759/cureus.26950

**Published:** 2022-07-17

**Authors:** Fatima Zahoor, Najia Ahmed, Ghazal Afzal

**Affiliations:** 1 Department of Dermatology, Pakistan Navy Station (PNS) Shifa Hospital, Karachi, PAK

**Keywords:** squamous cell carcinoma (scc), pd-1 inhibitor, targeted therapy, nivolumab, nail dystrophy

## Abstract

Nivolumab is a human immunoglobulin G4 (IgG4) monoclonal antibody that binds to the PD-1 receptor on T-cells and blocks its interaction with PD-L1 and PD-L2, releasing the PD-1 pathway-mediated inhibition of the immune response, including the anti-tumor immune response, resulting in decreased tumor growth. Here, we present a case of a 56-year-old lady with a diagnosis of squamous cell carcinoma (SCC) of the lip who presented with dystrophy of 20 nails, distal onycholysis, yellow-black discoloration of nail plates, painful paronychia with superimposed bacterial infection of big toes of both feet for three months. Few warty growths were also appreciated on big toes of both feet. She had undergone for her SCC 33 sessions of radiotherapy and 43 cycles of nivolumab 140mg for 60 minutes every two weeks. Following discontinuing this drug, the peri-ungual and nail bed inflammation improved, however nail plate dystrophy persisted. To our knowledge, the occurrence of nail dystrophy with nivolumab has never been reported before but it has been described with other targeted therapies.

## Introduction

Nivolumab is a targeted immunotherapy that acts by blocking human programmed death receptor-1 (PD-1) on T cells [1]. It is used in treatment of many cancers e.g., recurrent or metastatic squamous cell carcinoma of head and neck, metastatic melanoma, metastatic small cell lung cancer, advanced and metastatic cancers of esophagus, gastrointestinal tract and renal system. Its common side effects include fatigue, lymphocytopenia, decreased appetite and cough. Less common side effects include nausea, abdominal pain, constipation, fever, rash, immune-mediated hypothyroidism and hyperthyroidism, colitis, nephritis, pneumonitis and hepatitis. It also results in electrolyte imbalance. Nivolumab is a pregnancy category D drug and is hazardous to fetus so both men and women are advised not to conceive while taking this drug and five months after stopping this drug. These types of side effects have also been observed with other targeted therapies i.e. trastuzumab and alemtuzumab [2]. To our knowledge only one other case of nail dystrophy as a side effect of targeted therapy has been reported [3]. Therefore, we report the case of the lady who developed nail dystrophy as a side effect of nivolumab being taken for squamous cell carcinoma of the lip.

## Case presentation

**Figure 1:**
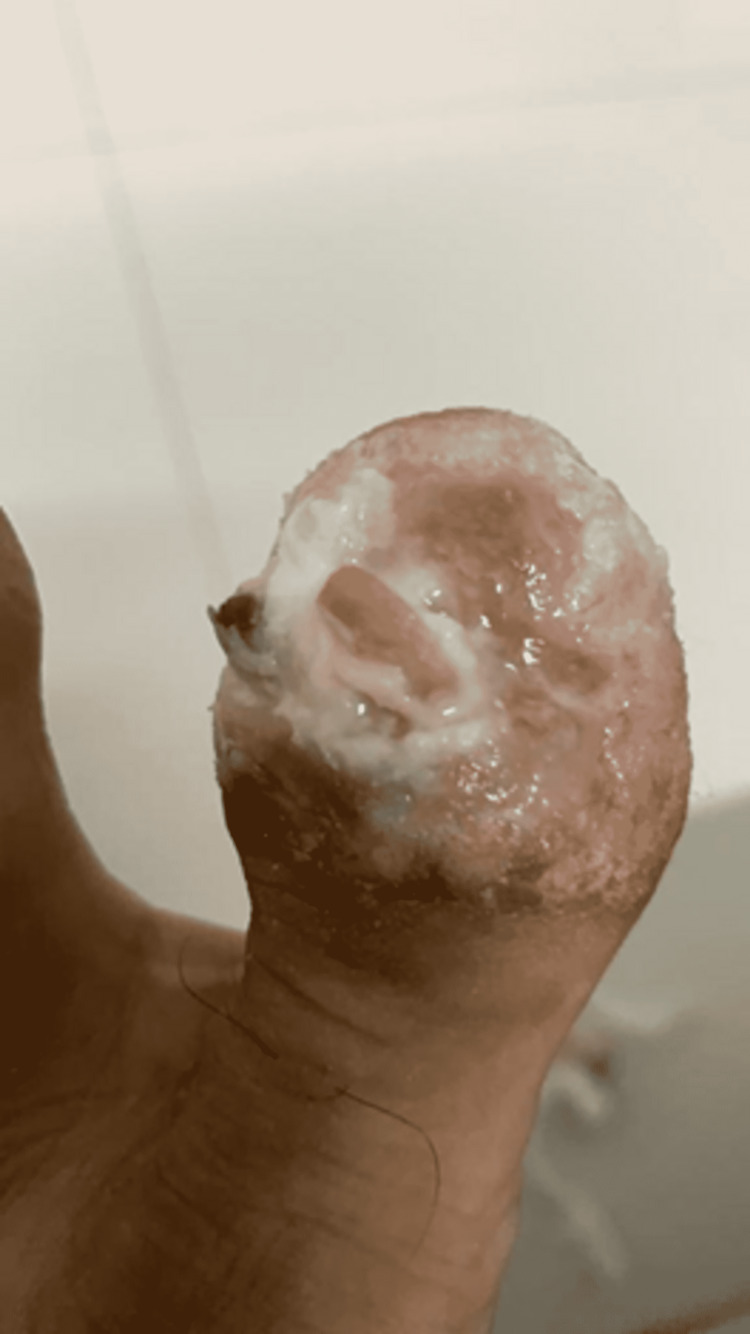
Nail bed inflammation with superimposed bacterial infection during treatment with nivolumab

A 52-year-old housewife, diagnosed case of squamous cell carcinoma of lip, taking IV nivolumab for two years presented in the Department of Dermatology at Pakistan Navy Station (PNS) Shifa Hospital, Karachi, with complaints of painful lesions in and around the big toenails of both feet along with blackish discoloration for three months. Physical examination revealed inflammation of nail fold of big toes of both feet along with yellow-black discoloration of nail plate, distal onycholysis and frank pus oozing (Figure 1).

**Figure 2:**
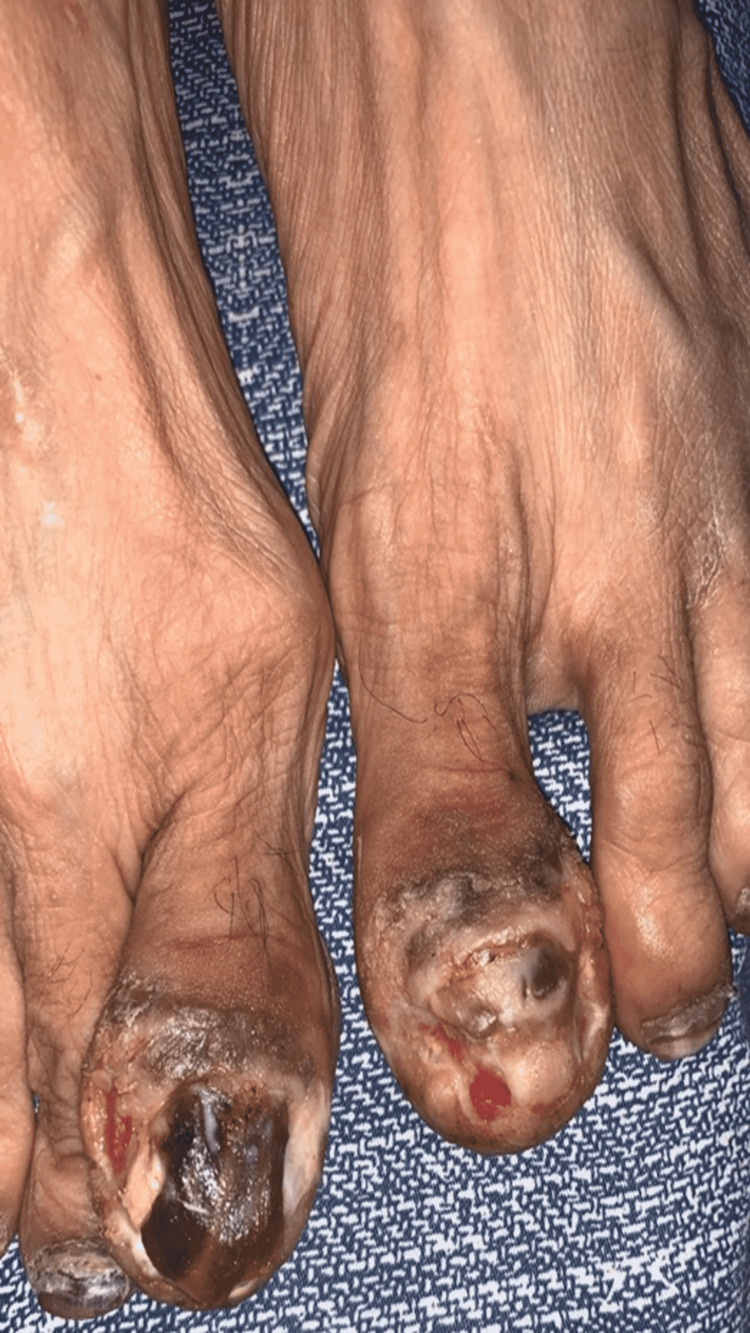
Resolution of nail bed inflammation, one month after stopping nivolumab

**Figure 3:**
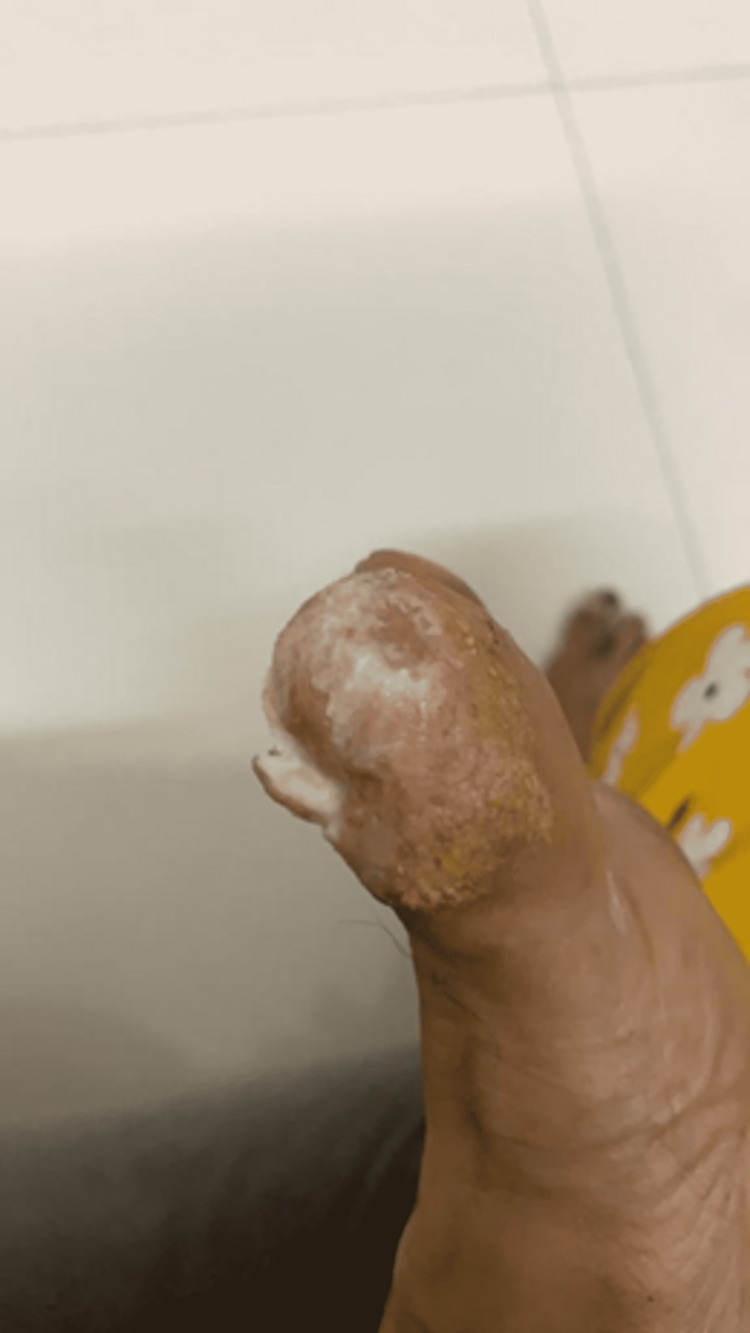
Lateral view of big toe of left foot after stopping the drug: Nail dystrophy persists but nail bed inflammation subsided

A few warty growths were also appreciated on the exposed part of the nail matrix. The other nails of both hands and feet were also discoloured. The patient was diagnosed with a case of squamous cell carcinoma of left side of lower lip in 2015. Resection of the tumor was done followed by 33 sessions of radiotherapy. After the resection surgery, the patient was on liquid diet as her mouth movements were restricted. Reoccurrence of tumor occurred in 2019 and patient was started on chemotherapy nivolumab 140 mg every two weeks. She had taken 43 cycles of nivolumab. She developed nail plate dystrophy of all 20 nails of hands and feet with superimposed bacterial infection and painful chronic paronychia of both big toes. The cycles of nivolumab were stopped after she developed nail changes. Her pus specimen was also taken for culture and sensitivity which came out positive for Staphylococcus aureus. Her haemoglobin (Hb) was 8.2 g/dl, platelet count of 155,000 per microliter of blood, erythrocyte sedimentation rate (ESR) was 55, chest X-ray, renal functions tests i.e. serum creatinine, urea, electrolytes and liver functions tests conjugated (direct bilirubin), SGOT (aspartate aminotransferase [AST]), SGPT (alanine aminotransferase [ALT]), alkaline phosphatase, total protein, albumin and globulin were normal. The patient was treated with antibiotic amoxicillin/clavulanic acid and analgesics and daily pyodine-Iodine dressing for two weeks and called for follow-up. After two weeks, the inflammation resolved and biopsy of the left big toe nail warty growth was taken. Histopathological examination revealed marked epidermal hyperkeratosis, neutrophilic crust formation, hypergranulosis, acanthosis and papillomatosis. The upper dermis showed mixed inflammatory infiltrate comprised of lymphocytes, plasma cells and neutrophils. There was no evidence of viral warts or granuloma or malignancy and the report suggested acute on chronic inflammation. The severe inflammation in and around the big toenails subsided about a month after stopping nivolumab. However, it reoccurred after the drug was started again and settled after stopping the drug (Figures 2, 3).

During the course of her treatment, she also experienced some other side effects of the drug like hypothyroidism for which her physician started tablet thyroxine 150mcg. She also had fatigue, nausea, decreased appetite and low sodium levels.

## Discussion

Nivolumab is a human monoclonal antibody that blocks the interaction between PD-1, PD-L1 and PD-L2. Binding of these ligands to the PD-1 receptor found on T cells, inhibits T cell proliferation and cytokine production, resulting in decreased tumor growth. Multiple side effects of targeted therapies have been reported [4]. The most common side effect encountered by patients taking nivolumab is fatigue, low sodium levels, lymphopenia, cough and decreased appetite. Less common are nausea, vomiting, diarrhea and electrolyte imbalance; our patient also suffered from multiple side effects and similar side effects have been reported with other targeted therapies. For instance, diarrhoea, anemia, and neutropenia were the most common with the patients taking pertuzumab and trastuzumab as defined by von Minckwitz et al. [5]. The patient described in this case report showed dystrophy of the nail plate of all 20 nails with distal onycholysis and severe inflammation in and around both big toenails. No such side effect has been reported before. However, in a recent study in 2021 a rare and unconventional side effect of nivolumab has been reported in a case report in which bluish-gray fingernail discoloration was induced due to use of nivolumab. According to that report the process through which nivolumab has caused this side effect remained to be clarified [6]. Another report, namely “Cutaneous adverse effects induced by nivolumab” also shed light upon nivolumab-induced cutaneous toxicity and claimed that specific cutaneous toxicity had been scarcely reported in the literature [7]. Immunotherapy is the newest in anticancer drug development that has immunomodulatory therapeutic antibodies, targeting inhibiting receptors expressed by T cell CTLA-4 and PD-1. They are used to treat advance stage cancer with metastasis or unresectable tumor such as melanoma and lung cancer [8]. Importantly, there was a case reported in a research article with preceding nivolumab treatment followed by vemurafenib who developed toxic epidermal necrolysis (TEN) [9]. Further, particular diagnosis and presentation necessitate drug exposure recording, imaging and skin biopsies. Meanwhile, according to another research side effects of immune checkpoint inhibitors such as ipilimumab, nivolumab, pembrolizumab and atezolizumab are caused by exacerbated immune responses against the individual’s own tissues. They occur in 64.2% of patients, and approximately 10-15% present with more severe conditions [10]. Immunotherapy has appeared as a superior method in management of different malignancies, especially advanced malignancies. Checkpoint inhibitors have resulted in dramatic success. However, their use can be accompanied by nonspecific immune activation leading to a myriad of autoimmune and autoinflammatory phenomena [11]. Meanwhile, according to another study, these drugs interfere with distinct mechanistic effectors central to pathogenesis and reduce the increasing nonspecific destruction caused by conventional anticancer chemotherapy, nonetheless, targeted therapies are still linked to a wide range of dermatologic adverse events because signaling routes are shared between malignant cells and normal mucocutaneous tissue [12]. In a nutshell, further research is warranted, as the specific mechanism through which the drug results in such side effects is yet to be known.

## Conclusions

Immunotherapy and targeted therapies have emerged as leading treatments for cancers and tumors. Nivolumab is the primary focus of this paper because the case that we present used this treatment and had side effects from its use. Later when the drug was stopped, the same effects waned. The focus of our study - severe inflammation of the nail bed and surrounding paronychia along with blackish discoloration of the nails - may be described as a rare side effect of nivolumab. Whereas, going through different literature reviews it is known that multiple effects occur through targeted therapies, including nivolumab. Therefore, an important outcome of this study is to display the effects of nivolumab and the controlling measures that resulted in reduction of the nail condition. However, the mechanism through which this occurs is yet to be determined and further research is warranted in order to fully understand the real cause behind the adverse effects.
